# Ultrasound-Assisted Extraction of Taro Leaf Antioxidants Using Natural Deep Eutectic Solvents: An Eco-Friendly Strategy for the Valorization of Crop Residues

**DOI:** 10.3390/antiox12101801

**Published:** 2023-09-26

**Authors:** Atalanti Christou, Nikolaos A. Parisis, Themistoklis Venianakis, Alexandra Barbouti, Andreas G. Tzakos, Ioannis P. Gerothanassis, Vlasios Goulas

**Affiliations:** 1 Department of Agricultural Sciences, Biotechnology and Food Science, Cyprus University of Technology, Lemesos 3603, Cyprus; 2 Department of Chemistry, Section of Organic Chemistry and Biochemistry, University of Ioannina, 45110 Ioannina, Greece; nparisis@uoi.gr (N.A.P.); vethemis@gmail.com (T.V.); atzakos@uoi.gr (A.G.T.); igeoth@uoi.gr (I.P.G.); 3 Department of Anatomy-Histology-Embryology, Faculty of Medicine, School of Health Sciences, University of Ioannina, 45110 Ioannina, Greece; abarbout@uoi.gr

**Keywords:** *Colocasia esculenta* L., taro leaves, natural deep eutectic solvents (NaDESs), response surface methodology (RSM), ultrasound-assisted extraction (UAE), waste valorization, green chemistry, eco-friendly extraction, polyphenols, antioxidants

## Abstract

*Colocasia esculenta* L. leaves are considered a by-product of taro cultivation and are discarded as environmental waste, despite their valuable phenolic composition. Their valorization to obtain value-added substances for medicinal, food, and cosmetic applications is the aim of the current work. An ultrasound-assisted extraction was developed for the environmentally friendly and sustainable isolation of taro leaf antioxidants using natural deep eutectic solvents (NaDESs). Among the utilized solvents, the NaDES based on betaine and ethylene glycol provided the best extraction efficiencies in terms of polyphenolic content and antioxidant activity. Multi-response optimization suggested a solvent-to-solid ratio of 10 mL g^−1^, a processing time of 60 min, an extraction temperature of 60 °C, and a water content of 33.8% (*w*/*w*) as optimal extraction parameters. Leaf extract obtained under these optimum operational parameters demonstrated a strong radical scavenging activity against 2,2-diphenyl-1-picrylhydrazyl (65.80 ± 0.87%), a high ferric reducing antioxidant power (126.62 ± 1.92 μmol TE g^−1^ sample), and significant protection against oxidative stress-induced DNA damage. The chromatographic characterization of the optimum extract revealed its richness in flavonoids (flavones and flavonols). The outcomes of the present study suggest that the proposed method could serve as a highly efficient and green alternative for the recovery of polyphenols from agricultural wastes.

## 1. Introduction

*Colocasia esculenta* (L.), commonly known as taro, is an annual herbaceous plant belonging to the Araceae family [1]. Despite its adaptation to tropical and subtropical regions (Africa, the Pacific region, and Asia), taro has long been cultivated in the Mediterranean and southern Europe [2,3]. In Cyprus, it has been integrated into the local cuisine and has been certified as a Protected Designation of Origin (PDO) product. This tropical species is mainly produced for its underground starchy tubers (mother corm and side cormels), which are considered an essential food for millions of people worldwide, and represents the 14th most cultivated vegetable around the world [1]. However, crop residues, namely, taro leaves, stems, and flowers, which represent the above-ground and largest part of the plant, remain unexploited [4]. Despite these plant parts being an excellent source of valuable components such as proteins, β-carotene, potassium, calcium, phosphorus, iron, riboflavin, thiamine, niacin, vitamin A, vitamin C, dietary fiber, and polyphenolic compounds, most of this generated biomass is directly discarded as waste to the environment, causing certain economic and environmental issues [2].

The exploitation of this agri-food waste material as a renewable and inexpensive source of natural antioxidants is an attractive option from an economic and environmental point of view. In particular, taro leaves and stems could be a remarkable source of flavonoids (mainly luteolin, apigenin, and chrysoeriol glucosides), as well as phenolic acids (mainly coumaric, gallic, and caffeic acid derivatives) [1,2,5,6]. Due to their radical scavenging ability, polyphenolic substances can serve as protective agents against oxidation and can further be used as a bioactive ingredient in food, cosmetics, and pharmaceutical formulations [7].

Traditionally, the isolation of phenolic substances from plant matrices usually relies on conventional solid–liquid extraction techniques, including maceration, percolation, and Soxhlet extraction [8]. These procedures require long processing times, high temperatures, large volumes of organic solvents and they have low extraction efficiency, high energy consumption resulting lower environmental friendliness [9]. In addition, the extracts obtained must undergo solvent removal and further purification before use due to solvent toxicity [10]. As modern society demands environmentally friendly processes, new extraction techniques referred to as green or clean technologies are designed to reduce or eliminate the use of toxic solvents, preserve the environment, and reduce energy consumption [11]. Among them, ultrasound-assisted extraction (UAE), microwave-assisted extraction (MAE), supercritical fluid extraction (SFE), and pressurized liquid extraction (PLE) demonstrate high extraction yields and high-quality extracts while reducing or eliminating the use of toxic solvents [12].

Natural deep eutectic solvents (NaDESs) are an emerging group of fluids that are considered a greener, safer, and more promising alternative to conventional organic solvents. They are entirely composed of natural components, mainly plant-based primary metabolites, such as amino acids, sugars, sugar alcohols, and organic acids [13]. They are prepared by simply mixing two or more components, one acting as a hydrogen bond donor (HBD) and the other as a hydrogen bond acceptor (HBA), in appropriate molar ratios to form eutectic mixtures that have a lower melting point (100 °C or lower) than their starting materials [14]. Depending on the nature and molecular proportions of their starting materials, NaDESs can exhibit a variety of physicochemical properties (density, viscosity, melting point, polarity, ionic conductivity, acidity, or alkalinity) that, in turn, determine their applicability [15]. These substances have many advantages over conventional solvents, including negligible vapor pressure, nonflammability, low toxicity, biocompatibility, exceptional solvation properties, and high recyclability, making them excellent solvents for the implementation of eco-friendly extraction strategies [16]. In addition, their natural components enable the direct use of NaDESs in food, cosmetic and pharmaceutical products, while their strong stabilizing power, resulting from the formation of hydrogen bonds between the solute components and NaDESs, protects the extracted molecules from oxidative degradation [13,17]. The literature has recently provided several examples of NaDES applications for the extraction of biologically active substances, especially polyphenols, from a variety of plant matrices and agrifood wastes, including red grape pomace, onions, olives, tomatoes, pomegranate, and orange peels, among others [15,18].

Given the richness of bioactive phytochemicals in taro leaves, the present study aims to develop an environmentally friendly, cost-effective, and sustainable extraction procedure for the effective recovery of taro leaf antioxidants. Thus, a green process was developed for the first time combining the benefits of the use of NaDESs and ultrasound radiation. To the best of our knowledge, there is no previous report regarding the green recovery of phenolic antioxidants from taroleaves using NaDESs coupled with UAE. To date, no studies have focused on optimizing the recovery of polyphenols from taro leaves, but have determined their phenolic composition [1,2,5,6,19,20]. Under this framework, sixteen different NaDESs were prepared and characterized. The most suitable NaDES for the recovery of polyphenols with antioxidant properties was used to develop a novel UAE method for the recovery of polyphenolic antioxidants from taro residues. UAE operational parameters, namely, the solvent-to-solid ratio, extraction time, extraction temperature, and water content in the NaDES, were optimized using response surface methodology (RSM).

The in vitro antioxidant activity of the optimum extract was determined using spectrophotometric assays (2,2-diphenylpicrylhydrazyl (DPPH), and ferric reducing antioxidant power (FRAP)) and by measuring the protective effect of the extract against H_2_O_2_-induced DNA damage. Its phenolic composition was characterized using both spectrophotometric (total phenolic content (TPC), total flavonoid content (TFC), total hydroxycinnamic acids (THA), and total flavonols (TF)) and chromatographic (ultra-performance liquid chromatography-quadrupole time-of-flight mass spectrometry, UPLC-QTOF-MS) methods of analysis. The outputs of the present work suggest that the by-products of taro cultivation can potentially be considered an important and readily available source of natural antioxidants.

## 2. Materials and Methods

### 2.1. Standards and Reagents

All chemicals were of analytical reagent grade. LC-MS grade water (H_2_O) and acetonitrile (ACN) were purchased from Supelco (Bellefonte, PA, USA). Ethylene glycol (EtGl), propylene glycol (PrGl), glycerol (Gly), citric acid (CA), d-(+)-glucose (Glc), sodium nitrite (NaNO_2_), sodium acetate trihydrate (C_2_H_3_NaO_2_·3H_2_O), and aluminum chloride (AlCl_3_) were obtained from Sharlau Chemie (Barcelona, Spain). The analytical standards of gallic acid, catechin, caffeic acid, quercetin, and trolox were obtained from Sigma-Aldrich (Steinheim, Germany). Folin-Ciocalteu reagent, sodium carbonate (Na_2_CO_3_), hydrochloric acid (HCl), formic acid, 2,2-diphenyl-1-picrylhydrazyl (DPPH), acetic acid, 2,4,6-tris(2-pyridyl)-s-triazine (TPTZ), d-(-)-Fructose (Fru), Sucrose (Suc), choline chloride (ChCl), betaine anhydrous (Bet), and l-(+)-lactic acid (LA) were also acquired from Sigma-Aldrich (Steinheim, Germany). Ethanol (EtOH) and sodium hydroxide (NaOH) were purchased from Merck (Darmstadt, Germany), while methanol (MeOH) and iron(III) chloride hexahydrate (FeCl_3_⋅6H_2_O) were obtained from Honeywell (Charlotte, NC, USA).

### 2.2. Preparation and Characterization of NaDESs

#### 2.2.1. Preparation of NaDESs

Sixteen different NaDESs were prepared based on ChCl and Bet as the HBAs in combination with polyols (EtGl, PrGl, Gly), acids (LA and CA), and sugars (Fru, Glc, Suc) as the HBDs (Table 1). The NaDESs were prepared by stirring and heating their components (HBA and HBD), in a defined molar ratio, at 80 °C in sealed flasks until a transparent and colorless liquid was obtained (between 30 and 120 min) [21]. The obtained eutectic mixtures were allowed to cool and then mixed with water (20%, *w*/*w*) to reduce their viscosity and increase their solvation power. Finally, they were stored in sealed vials, in the dark, at room temperature, in a desiccator.

#### 2.2.2. Characterization of NaDESs

##### Viscosity

The rheological behavior of the prepared NaDESs containing 20 and 40% (*w*/*w*) water was determined using a Brookfield LVDV-E Viscometer (Brookfield Engineering Laboratories, Middleboro, MA, USA). All viscosity measurements were operated at 25 °C and rotation rates from 5 to 100 rpm using the S03 spindle.

##### pH

NaDESs containing 20% and 40% water (*w*/*w*) were characterized according to their pH using a digital pH meter (edge^®^blu, Hanna Instruments, Woonsocket, RI, USA). All pH measurements were performed at 25 °C.

##### Fourier-Transform Infrared Spectroscopy (FTIR)

FTIR spectra of the prepared NaDESs (before water addition) and their individual components were recorded using a Shimadzu IRPrestige-21 spectrophotometer (Shimadzu, Tokyo, Japan) to obtain information on possible interactions, structural changes, and hydrogen bond formation in the NaDESs. The spectra were acquired in the range of 4000–600 cm^−1^, with a spectral resolution of 32 cm^−1^ and an accumulation of 4 scans. To collect the spectra of the solid samples, the KBr pellet method was utilized.

### 2.3. Plant Material and Sample Pre-Treatment

Taro (*Colocasia esculenta*) leaves, carrying the PDO certification, were collected from Sotira village (Famagusta, Cyprus, 35°01′18.8″ N 33°56′10.4″ E) at the end of October after the harvest of corms. The PDO residue was kindly provided by local producers. Immediately after their collection, taro leaves were transferred to the laboratory, washed with distilled water, and dried in an oven at 40 °C for 48 h. The dry material was then powdered using an electric mill and stored until further use.

### 2.4. Optimization of Ultrasound-Assisted Extraction (UAE) Using Response Surface Methodology (RSM)

UAE of phenolics was performed by use of a 250 W power and 35 kHz frequency ultrasonic bath (UCI-50, 35 KHz, Raypa-R. Espinar, S.L., Terrassa, Spain). For the extraction, 0.2 g of leaf powder were transferred to a series of Falcon centrifuge tubes and mixed with different volumes (2, 4, and 6 mL) of NaDES solutions containing different contents of water (20, 30, and 40% *w*/*w*). The samples were mixed well for 1 min (vortex) and then placed in an ultrasonic bath for extraction. The obtained suspensions were exposed to acoustic waves at various temperatures (20, 40, and 60 °C) and for varying periods of time (20, 40, and 60 min) according to the developed experimental design or screening study. After the ultrasound treatment, the obtained mixtures were allowed to cool and were then centrifuged at 2500 rpm for 20 min. The supernatants were collected and stored at 4 °C for further analysis. All extracts were prepared in triplicate.

RSM based on a Box–Behnken design (BBD) was employed to investigate the effects of independent variables on the responses and to determine the optimal UAE operational parameters. In particular, a three-level and four-factor BBD was applied to evaluate the effect of solvent-to-solid ratio (A), extraction time (B), extraction temperature (C), and water content (D) on the responses, namely, total phenolic content (TPC, Y_1_), total flavonoid content (TFC, Y_2_), total hydroxycinnamic acids (THA, Y_3_), total flavonols (TF, Y_4_), DPPH inhibition (Y_5_), and ferric reducing antioxidant power (FRAP, Y_6_). The independent variables in their coded and actual levels are presented in Table 2. The complete experimental design consisted of 29 combinations including five replicates at the central point. All measurements were performed in triplicate and in a randomized order to reduce bias (Table 3). The mean values of all dependent parameters, obtained from the triplicate analysis of responses, were fitted to the quadratic polynomial model described in Equation (1), except for the THA response, which was best described by the linear model in Equation (2). Analysis of variance (ANOVA) was conducted to investigate the effect of linear, interaction, and quadratic regression coefficients and to determine the validity of the models. Three-dimensional (3D) response surface plots were constructed based on the developed regression models to visualize the relationship between dependent variables and independent factors. Design Expert software (trial version 11.0, Stat-Ease Inc., Minneapolis, MN, USA) was utilized for the design of experiments, model building, and data interpretation.

(1) $$Y=\beta_{0}+\sum_{i=1}^{n}\beta_{\iota }x_{i}+\sum_{\begin{array}{c} i=1\\ j>1 \end{array}}^{n-1}\sum_{j=2}^{n}\beta_{ij}x_{i}x_{j}+\sum_{i=1}^{n}\beta_{ii}{x_{i}}^{2}$$

(2) $$Y=\beta_{0}+\sum_{i=1}^{k}\beta_{\iota }x_{i}$$

where *β_0_* is the constant coefficient, *β_i_*, *β_ii_*, and *β_ij_* are the regression coefficients for linear, quadratic, and interaction terms, respectively, *x_i_* and *x_j_* represent the independent variables, and *k* is the number of variables (*k* = 4).

### 2.5. Phytochemical Analysis

#### 2.5.1. Determination of Total Phenolic Content (TPC) of Colocasia Leaf Extracts

The TPC of Colocasia leaf extracts was determined using the 96-well microplate Folin-Ciocalteu colorimetric method described by Bobo-García et al. [22] with slight modifications. In brief, 20 μL of suitably diluted extract solution was mixed with 100 μL of Folin-Ciocalteu reagent (1:4, *v*/*v*, diluted with distilled water) in a flat-bottom 96-well microplate, and the resulting mixture was shaken for 1 min. The mixture was allowed to stand for 4 min, and then 75 μL of saturated sodium carbonate solution (100 g L^−1^) was added followed by the addition of 100 μL of 4% (*w*/*v*) sodium hydroxide solution. The obtained mixture was shaken for 1 min and then allowed to stand in the dark at room temperature for 2 h. The absorbance of the reaction mixture was then measured at 750 nm using a Thermo Scientific Multiskan GO spectrophotometer (ThermoFisher Scientific, MA, USA). Gallic acid was used as a reference standard (y = 2.7028x + 0.0603, R² = 0.997), and total phenolics were expressed as mg of gallic acid equivalents (GAE) per g of dry sample.

#### 2.5.2. Determination of Total Flavonoid Content (TFC) of Colocasia Leaf Extracts

The TFC of taro leaf extracts was evaluated using the aluminum chloride colorimetric method as described by Goulas et al. [23]. Briefly, 25 μL of extract solution was mixed with 100 μL of distilled water and 10 μL of a 50 g L^−1^ sodium nitrite solution in a flat-bottom 96-well microplate. After waiting for 5 min, 15 μL of aluminum chloride solution (100 g L^−1^) was added to the reaction mixture. Then after another 6 min, aliquots of 50 μL of sodium hydroxide solution (1 M) and 50 μL of distilled water were added and the reaction mixture was shaken for 30 s. The absorbance of the resulting mixture was measured at 510 nm using a Thermo Scientific Multiskan GO spectrophotometer. Catechin was used as a standard for calibration (y = 1.6348x + 0.0384, R² = 0.9991), and the TFC values were expressed as mg of catechin equivalents (CE) per g of dry sample.

#### 2.5.3. Determination of Total Hydroxycinnamic Acids (THA) and Total Flavonols (TF) of Colocasia Leaf Extracts

The content of THA and TF was determined spectrophotometrically according to the method described by Goulas et al. [24] with minor modifications. Determination of THA and TF contents was performed by mixing 20 μL of suitably diluted extract solution with 20 μL of 0.1% (*v*/*v*) HCl solution (in ethanol) and 160 μL of 2% (*v*/*v*) HCl ethanolic solution. The absorbances of the resulting mixtures were measured at 320 and 360 nm for the determination of THA and TF, respectively, using a Thermo Scientific Multiskan GO spectrophotometer. Caffeic acid (y = 5.0165x + 0.276, R² = 0.9985) and quercetin (y = 2.5104x + 0.1247, R² = 0.9997) were used to construct the respective calibration curves, and the results obtained were expressed as mg of caffeic acid equivalents (CAE) per g of dry sample for THA and as mg of quercetin equivalents (QE) per g of dry sample for TF.

#### 2.5.4. Ultra-Performance Liquid Chromatography Coupled to Electrospray Ionization-Quadrupole-Time-of-Flight Mass Spectrometry (UPLC/ESI-QTOF-MS) to Determine the Phytochemical Profile of Colocasia Leaf Extract

The phytochemical profile of the extract obtained under the optimal UAE conditions was elucidated by UPLC/ESI-QTOF-MS. Before its injection into the chromatographic system, the NaDES extract was suitably diluted and filtered through a 0.2-μm RC filter. A comprehensive literature search on the polyphenolic profile of taro leaves was previously conducted, and a library of structural compounds was generated based on the compounds formerly identified. Compounds were characterized based on retention times, mass data obtained by ESI-QTOF-MS, and fragmentation pattern, compared with reference standards and an in-house library.

Chromatographic separation of polyphenolic compounds was performed on an ACQUITY iClass Plus UPLC system equipped with a thermostatic autosampler set at 6 °C, using an Acquity T3-HSS C_18_ column (100 mm × 2.1 mm i.d., 1.7 μm particle size) (Waters, MA, USA), which was kept at 45 °C. The mobile phase consisted of 0.1% (*v*/*v*) formic acid (solvent A) and ACN containing 0.1% (*v*/*v*) formic acid (solvent B) and was pumped through the column at a flow rate of 0.4 mL min^−1^. The gradient elution program was as follows: 0.00–0.07 min, 1% B; 0.07–10.00 min, 1–100% B; 10.00–12.67 min, 100% B; 12.67–12.73 min, 100–1% B; 12.73–15.00 min, 1% B for column equilibration. The sample injection volume was 2 μL.

The Xevo G2-X2 Q-ToF mass spectrometer (Waters, Manchester, UK) was coupled to the ACQUITY UPLC iClass Plus system via an electrospray ionization (ESI) interface. The Xevo G2 Q-TOF mass spectrometer was operated in negative ESI polarity mode. Typical source conditions for maximum intensity of precursor ions were as follows: capillary voltage, 1.0kV; sample cone, 40 V; source temperature, 120 °C; desolvation temperature 550 °C; cone gas flow rate, 20 L h^−1^; and desolvation gas (N_2_) flow rate, 1000 L h^−1^. All analyses were performed using LockSpray, which ensured accuracy and reproducibility. Leucine–enkephalin (5 ng mL^−1^) was used as the lockmass, generating a reference ion in negative mode at *m*/*z* 554.2620, and introduced at 10 μL min^−1^ for accurate mass acquisition at a 60 s interval for 0.250 s. Data acquisition was achieved using MS^E^, which has two separate scan functions that are programmed with independent collision energies: Function 1 (low energy): 50–1200 mass-scan range; 0.2 s scan time; 4 eV collision energy; and Function 2 (high energy): 50–1200 mass-scan range; 0.2 s scan time; collision energy ramp of 25–45 eV. Acquiring data in this manner provided information on both intact precursor ions and fragment ions simultaneously. Data were acquired and processed using the UNIFI software platform (version 1.9.4.053, Waters MS Technologies, Manchester, UK). In-house phytochemical libraries were used for the identification of the compounds.

### 2.6. Antioxidant Capacity

#### 2.6.1. DPPH Radical Scavenging Activity

The radical scavenging ability of the extracts was measured from the bleaching of the purple-colored methanolic solution of DPPH following the procedure described by Roseiro et al. [25]. Briefly, extract solutions were appropriately diluted (25 mg sample mL^−1^) and mixed with 975 μL of freshly prepared DPPH methanolic solution (60 μM) or extraction solvent (blank). The samples were then vigorously shaken and incubated in the dark at room temperature for 30 min. The decrease in absorption of the DPPH solution was then measured at 517 nm using a Thermo Scientific Multiskan GO spectrophotometer. The DPPH radical scavenging activity (%) of the extracts was calculated according to the following equation:

(3) $$\%\;DPPH\;inhibition=\left(\frac{{Abs}_{b}-{Abs}_{e}}{{Abs}_{b}}\right)\times 100$$

where *Abs_b_* is the absorption of the blank (extraction solvent) and *Abs_e_* is the absorption of the investigated extract solution.

#### 2.6.2. FRAP Assay

The antioxidant potential of the extracts was also determined by the FRAP assay according to the method described by Benzie et al. [26]. For the preparation of the FRAP reagent, 300 mM acetate buffer (pH = 3.6), 10 mM TPTZ solution in 40 mM HCl, and 20 mM FeCl_3_ aqueous solution were mixed in the ratio of 10:1:1 (*v*/*v*/*v*) and then incubated at 37 °C before use. An aliquot of each diluted extract (150 μL) was then reacted with the FRAP solution (950 μL) and the resulting mixture was allowed to stand in the dark for 30 min. Readings of the colored product were then taken at 593 nm using a Thermo Scientific Multiskan GO spectrophotometer. The results were expressed as μmol of Trolox equivalents (TE) per gram of dry sample using a Trolox standard calibration curve (y = 0.0013x + 0.0906, R² = 0.9999).

### 2.7. Protective Effect against H_2_O_2_-Induced DNA Damage

#### 2.7.1. Cell Culture and Treatment

Jurkat cells (ATCC; clone A6-1) were grown in RPMI 1640 containing 10% *v*/*v* heat-inactivated FBS, 2 mM glutamine, 100 U mL^−1^ penicillin, and 100 μg mL^−1^ streptomycin, in 5% CO_2_ at 37 °C. Cells in the log phase were harvested, seeded in 96-well plates at the density of 1.5 × 10^6^ cells per ml (150,000 cells per well), and left for 1 h under standard culturing conditions before further treatment. Cells were then pre-incubated for 30 min with increasing concentrations of the taro extract before being exposed for 15 min to oxidative stress conditions in the form of H_2_O_2_. H_2_O_2_ was continuously generated (~10 μM H_2_O_2_ per min) by the action of the glucose oxidase enzyme (0.6 μg mL^−1^) which was added directly to the culture medium [27]. After the indicated treatments, cells were collected and checked for viability by trypan blue exclusion, before further analysis.

#### 2.7.2. Single-Cell Gel Electrophoresis (Comet Assay)

The alkaline comet assay for the estimation of single-strand DNA breaks was performed as previously described [27,28,29]. In brief, after washing, cells were suspended in 1% (*w*/*v*) low-melting-point agarose in phosphate-buffered saline and pipetted onto superfrosted glass microscope slides, which were then precoated with a layer of 1% (*w*/*v*) normal melting-point agarose. The agarose was allowed to set at 4 °C and subsequently, the slides were immersed in a cold lysis solution containing 2.5 M NaCl, 100 mM EDTA, 10 mM Tris, pH 10, 1% (*v*/*v*) Triton X-100 at 4 °C for 1 h to dissolve cellular proteins and lipids. After completion of lysis, the slides were placed in a horizontal electrophoresis tank containing ice-cold unwinding solution (0.3 M NaOH, 1 mM EDTA, pH~13) and kept at 4 °C for 40 min to allow DNA strand separation (alkaline unwinding). Electrophoresis was performed for 25 min in the same solution at 25 V (1 V/cm) and 300 mA. After electrophoresis, the slides were washed 3 times in ice-cold 0.4 M Tris, pH 7.5.

Subsequently, nucleoids were stained with Hoechst 33342 (10 mg/mL) and examined under a UV microscope with a 490 nm excitation filter at a magnification of X400. DNA damage was not homogeneous, and visual scoring was based on the characterization of 100 randomly selected nucleoids. The comet-like DNA formations were categorized into five classes (0, 1, 2, 3, and 4), representing an increasing extent of DNA damage visualized as a “tail”. Each comet was assigned a value according to its class. Accordingly, the overall score for 100 comets ranged from 0 (100 comets in class 0) to 400 (100 comets in class 4). In this way, the overall DNA damage of the cell population can be expressed in arbitrary units.

### 2.8. Statistical Analysis

All experimental assays were performed in triplicate. The results obtained were expressed as mean values ± standard deviation (SD). The means were compared and statistical differences were obtained through one-way ANOVA followed by Duncan’s multiple range test at a 95% confidence level. The differences between individual means were considered significant at *p* < 0.05.

In the screening study of the NaDESs, the mean values of the obtained data set were subjected to pattern recognition analysis. The data set to be treated consisted of a 17 × 6 matrix, in which rows represented the extraction solvents (17 solvents) and the columns the TPC, TFC, THA, TF, DPPH, and FRAP values. Prior to multivariate analysis, the data matrix was mean-centered and scaled to unit variance to standardize the statistical significance of all monitored responses. PCA, as an unsupervised pattern recognition technique, was applied to the data to reduce data dimensionality and to identify any existing clustering of solvents based on their extraction efficiency and initial components. All the afore-mentioned statistical analyses were performed using RStudio statistical software (version 1.3.1073).

## 3. Results and Discussion

### 3.1. NaDES Characterization

In the present study, sixteen different NaDESs were prepared based on ChCl and Bet as the HBAs in combination with EtGl, PrGl, Gly, LA, CA, Fru, Glc, and Suc as the HBDs (Table 1). Due to the high viscosity of the freshly prepared NaDESs, adding water was deemed necessary to adjust their viscosity, facilitate their application, and in some cases obtain a stable homogeneous material (for sugar-based NaDESs). In addition, water helps to break the surface tension, allowing a significant reduction in viscosity without disrupting the interactions between the NaDES components The addition of water was performed at two concentration levels (20 and 40%, *w*/*w*) and the physicochemical properties of the diluted NaDESs were determined (Table 1).

Viscosity is an essential characteristic of an NaDES that must be controlled, as highly viscous solvents hinder mass transfer, negatively affecting the extraction of bioactive substances. As demonstrated in Table 1, the viscosity of the prepared NaDESs varies significantly depending on their composition. In particular, the viscosity of the prepared NaDESs containing 20% (*w*/*w*) water was in the range of 35–17360 cP, while the addition of water (40%, *w*/*w*) significantly reduced the viscosity values in the range of 24–78 cP. The addition of water, therefore, can be evaluated as a positive feature in terms of favorable extraction applications. Comparing Bet- and ChCl-based NaDESs, the former demonstrated higher viscosity values, probably due to the formation of stronger hydrogen bonding interactions due to the presence of the carboxylate group. As far as HBDs are concerned, sugar-based NaDESs demonstrated the highest viscosities, while alcohol-based solvents had the lowest viscosity values. The high viscosity of NaDESs is mainly attributed to the large number of hydrogen bonds developed between HBD and HBA resulting in the loss of molecular mobility. In addition, the chemical structure of the starting components affects the viscosity of the prepared NaDESs; the longer the chain length in their structures, the higher the viscosity of the final solvent [30]. Among the sugar-based HBDs, sucrose had the highest viscosity, which may be due to its characteristics as a disaccharide that allows it to form more hydrogen bonds compared with the monosaccharides fructose and glucose [31]. Regarding the polyols, the viscosity increases with the number of hydroxyl groups in their structure [32].

pH is another factor that significantly affects the recovery of polyphenolic substances. According to the literature, polyphenols are more easily extracted when they are neutrally charged, that is, when the pH of the solvent is lower than their pKa values [33]. As demonstrated in Table 1, the initial components used to prepare the NaDESs significantly affect the pH of the final solvents. As expected, NaDESs with organic acids as HBDs (LA and CA) presented the lowest pH values, followed by the sugar-based and the alcohol-based NaDESs, which is in accordance with the literature data [34].

**Figure 1:**
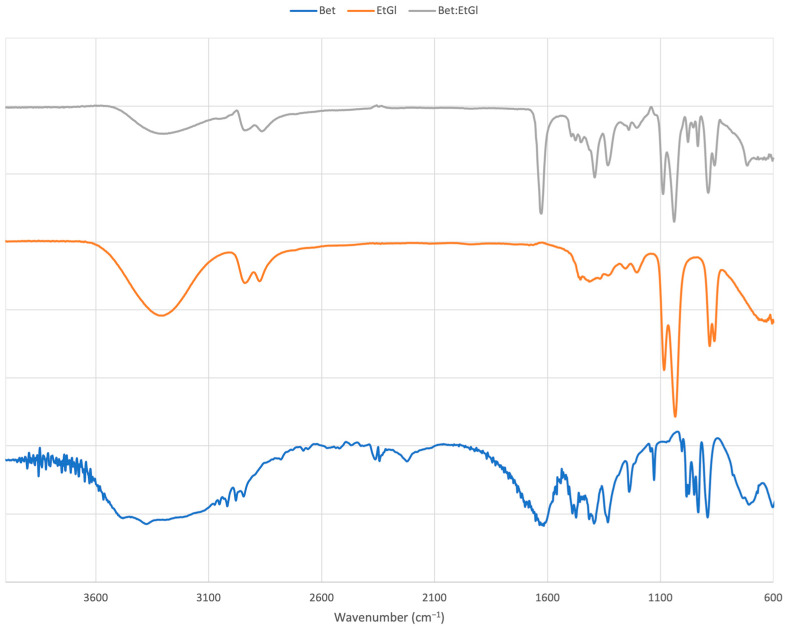
Fourier-transform infrared spectra of Bet, EtGl, and the prepared NaDES. The blue spectrum represents the hydrogen bond acceptor (HBA) (Bet), the red spectrum represents the hydrogen bond donor (HBD) (EtGl) and the grey spectrum represents the NaDES.

The formation of the eutectic mixtures was also confirmed by the use of FTIR spectroscopy. As a representative example, Figure 1 illustrates the spectra obtained for Bet, EtGl, and the formed NaDES. The FTIR spectrum of Bet has the characteristic bands of C-N asymmetric and symmetric stretching at 3486.54 and 3389.92 cm^−1^, respectively, and the characteristic band for the asymmetric stretching vibration of the carboxylate group at 1610.56 cm^−1^. In the EtGl IR spectrum, a broad band at 3304.06 cm^−1^ was observed which is related to the stretching vibrations of the hydroxylated groups in the EtGl structure. An increase in the bandwidth of hydroxylated groups was observed in the case of NaDES, indicating the formation of hydrogen bonds between the NaDES components. According to the literature, the interaction of NaDES components is established by the formation of hydrogen bonds between the carboxylate group (COO^−^) in Bet and the hydroxyl group (OH) of the polyol [35]. This interaction modifies the stretching vibration of the carbonyl group in Bet and the O-H bond in polyol, resulting in a wavelength shift from 1604.04 cm^−1^ (Bet) to 1627.92 cm^−1^ (BetEtGl), and from 3304.06 cm^−1^ (EtGl) to 3286.70 cm^−1^ (BetEtGl), respectively. Similarly, the FTIR analysis verified the formation of hydrogen bonds in all the prepared NaDESs.

### 3.2. Screening the Efficiency of NaDESs in the Recovery of Polyphenols from Taro By-Products

The polyphenol extractability of the sixteen prepared NaDESs (Table 1), which were grouped into three categories, polyol-, acid-, and sugar-based NaDESs, was assessed using the following extraction parameters: solvent-to-solid ratio = 20 mL g^−1^, processing time = 40 min, extraction temperature = 40 °C, and water content = 40%, *w*/*w*. The results demonstrated that the increase in water content from 20% to 40% (*w*/*w*) in the NaDESs reduces the solvent viscosity and increases extraction medium polarity, but it results in a reduction in extraction efficiency due to the weakening or breaking of the intermolecular hydrogen bond structure of NaDES components. Therefore, no water content greater than 40% (*w*/*w*) was used in any experiment in the present study. For comparison purposes, aqueous ethanol (40%, *v*/*v*) was chosen as the reference solvent, given its wide application as a conventional green solvent and the abundance of relevant scientific data in the literature. The recovery of taro polyphenols using different NaDESs was evaluated in terms of polyphenolic composition and antioxidant activity of the extracts using an array of assays, namely, TPC, TFC, THA, TF, DPPH, and FRAP.

**Figure 2:**
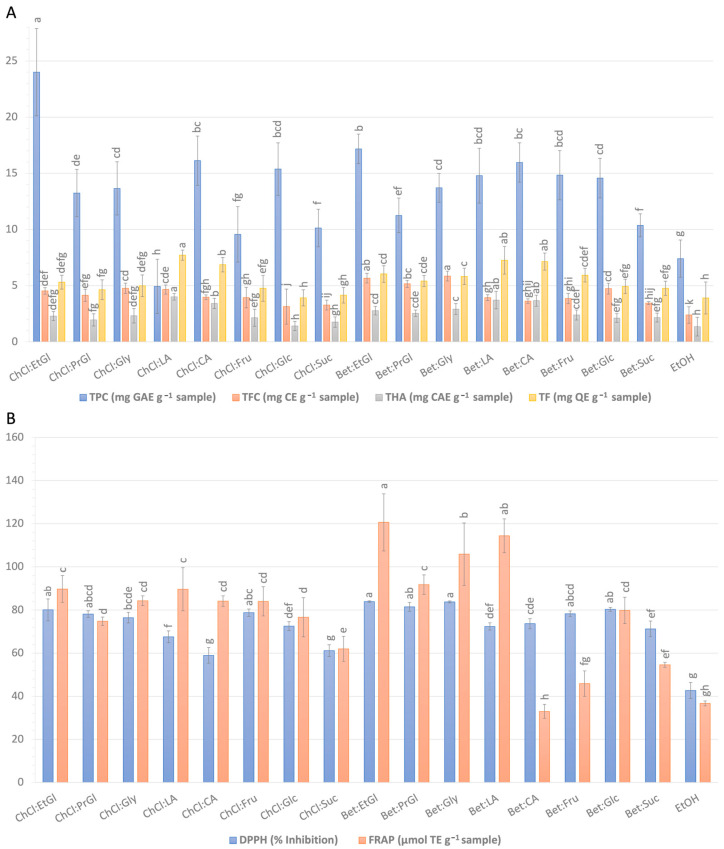
The effect of NaDES composition on (**A**) the total content of extracted polyphenolic components (TPC, TFC, THA, TF) and (**B**) the antioxidant activity (% inhibition of DPPH radical, FRAP) of the extracts. Different lowercase letters for each colored bar indicate significant differences (*p* < 0.05) according to Duncan’s multiple range test.

Figure 2 presents the total content of extracted polyphenolic components (TPC, TFC, THA, TF) and the antioxidant activity (% inhibition of DPPH radical, FRAP) of the extracts as a function of the different solvent types. As observed, all the NaDESs exhibited higher extraction yields than aqueous EtOH, indicating their superiority over a commonly used solvent systems. At the same time, the extraction efficiency of NaDESs strongly depends on the type of solvents and, in particular, on the combination of HBD and HBA. The extraction yields of TPC, TFC, THA, and TF obtained using NaDESs ranged from 4.94 to 24.00 mg GAE g^−1^ sample, from 3.13 to 5.85 mg CE g^−1^ sample, from 1.41 to 4.01 mg CAE g^−1^ sample, and from 3.92 to 7.71 mg QE g^−1^ sample, respectively. Significant differences were also observed in the antioxidant activities of the extracts obtained using NaDESs, exhibiting DPPH radical inhibitory activities in the range of 58.96–83.90% and FRAP values ranging from 32.99 to 120.58 μmol TE g^−1^ sample.

**Figure 3:**
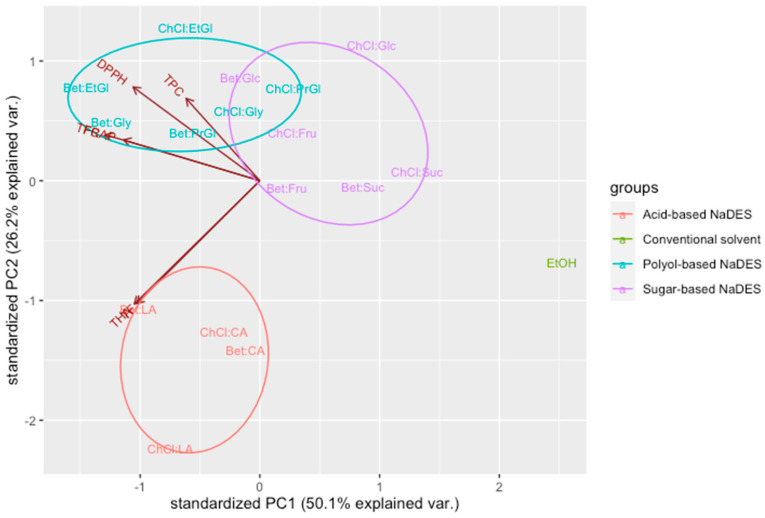
Principal component analysis (PCA) biplot demonstrating the distribution of extracts obtained using the prepared NaDESs and conventional solvent (40%, *v*/*v*, ethanol (EtOH)).

PCA was then applied to better visualize the differences in the extractability of antioxidants from taro leaves using different types of solvents (Figure 3). The first two principal components (PCs) were able to explain 76.3% of the total variability of the system, with PC_1_ accounting for 50.1% and PC_2_ for 26.2%. In the obtained PCA biplot (Figure 3), three clear clusters of samples were observed. The first group, which consists of polyol-based NaDESs, was located in the upper and left part of the diagram, indicating the high TPC, TFC, DPPH, and FRAP values of the respective solvents. The second group of NaDESs, which are the acid-based ones, were able to extract hydroxycinnamic acids and flavonols more efficiently. Sugar-based NaDESs were concentrated in the right part of the plot, demonstrating their poor extractive potential compared with the other eutectic mixtures. Aqueous ethanol was located in the right part of the plot, far away from the clusters of NaDESs, highlighting its low efficiency in the extraction of antioxidant components.

Among the examined NaDESs, polyol-based solvents provided the highest polyphenol yields and antioxidant activities, followed by the acid-based ones. The NaDESs prepared using sugars as HBDs proved to be the least effective eutectic mixtures in terms of antioxidant recovery. This is consistent with findings in the literature reporting the superiority of polyol-based NaDESs over other eutectic mixtures [36]. NaDES extraction efficiency mainly depends on its hydrogen bond formation capacity. Basically, NaDESs are composed of HBAs and HBDs, which can form hydrogen bonds with phenolic substances, increasing the dissolution capacity of target analytes. Other factors such as solvent acidity and polarity may affect the extraction efficiency of the prepared NaDES. Viscosity is also a critical factor that can affect mass transfer and the cavitation phenomenon during ultrasonic extraction, thus having a significant effect on the efficiency of the extraction solvent [9]. Polyol-based NaDESs appear to have a polarity similar to that of the target constituents and relatively low viscosity, thus allowing the formation of a strong hydrogen bond network with the target analytes [37]. As far as acid-based NaDESs are concerned, their acidic nature may have contributed to the disruption of the cell wall structure, thus facilitating the release of the target analytes from the plant matrix into the extraction medium [38]. On the other side, the high viscosity of sugar-based NaDESs led to mass transfer limitations by reducing the interactions between the extraction solvent and target analytes, resulting in decreased extraction efficiencies [39].

Comparing Bet- and ChCl-based NaDESs, the former exhibited higher extraction efficiencies in almost all cases, probably due to the formation of stronger hydrogen bond interactions with target substances. Considering their higher extraction efficiencies and lower cytotoxicity compared with solvents based on ChCl [35], Bet-based NaDESs were selected for the subsequent optimization study. When Bet-polyol NaDESs were compared, the eutectic mixture prepared using EtGl as the HBD was subjected to further optimization as it was the one that gave the highest concentration of polyphenols and antioxidant capacity. The steric hindrance of PrGl probably led to weaker interactions of the respective solvents with target analytes, resulting in lower extraction yields compared with EtGl- and Gly-based NaDESs [40]. The improved extraction performance of Bet:EtGl over Bet:Gly can be attributed to its lower viscosity resulting in better extraction of phenolics. Based on the results obtained, Bet:EtGl was selected as the solvent for the following optimization experiments.

### 3.3. Optimization of UAE Using RSM

#### 3.3.1. Model Fitting and Statistical Analysis

Once the most suitable solvent for extracting antioxidants from taro leaves was determined, RSM based on BBD was conducted to assess the influence of UAE processing parameters on the extractability of the target components and determine the optimal experimental conditions for polyphenols recovery. The impact of four independent variables, namely, solvent-to-solid ratio, extraction time, extraction temperature, and water content, at their most promising levels based on preliminary studies and existing literature, on the efficacy of UAE of taro antioxidants was investigated through a complete experimental design. The experimental results of the investigated responses (dependent variables), namely, TPC, TFC, THA, TF, DPPH, and FRAP, obtained under the different sets of UAE parameters are presented in Table 3. The results were in the range of 15.90–20.20 mg GAE g^−1^ sample for TPC, 5.49–7.62 mg CE g^−1^ sample for TFC, 2.28–4.16 mg CAE g^−1^ sample for THA, 5.18–8.76 mg QE g^−1^ sample for TF, 31.48–60.22% for DPPH, and 78.08–131.80 μmol TE g^−1^ sample for FRAP.

Regression analysis was applied to the experimental results, acquired in a randomized order, to obtain the mathematical equations describing the relationships between the system responses and independent variables. TPC, TFC, TF, DPPH, and FRAP responses were fitted to second-order polynomial equations, while THA was best described by a linear regression model. The generated polynomial equations, in terms of the coded factors, are given below.

(4) $$\begin{array}{c} TPC=18.21+0.37\;A+0.22\;B+0.83\;C+0.65\;D-0.30\;AB-1.06\;AC-0.05\;AD-0.94\;BC+0.25\;BD+\\ 0.16\;CD-0.02\;A^{2}+0.18\;B^{2}+0.14\;C^{2}-0.88\;D^{2} \end{array}$$

(5) $$\begin{array}{c} TFC=6.49+0.31\;A+0.35\;B+0.51\;C+0.13\;D+0.05\;AB+0.03\;AC+0.01\;AD-0.02\;BC-0.1\;BD+\\ 0.03\;CD+0.25\;A^{2}+029\;B^{2}+0.01\;C^{2}-0.22\;D^{2} \end{array}$$

(6) THA=3.27−0.53 A+0.35 B+0.28 C+0.28 D

(7) $$\begin{array}{c} TF=6.40-0.82\;A+0.40\;B+0.50\;C+0.32\;D-0.43\;AB-0.08\;AC+0.11\;AD-0.08\;BC+0.24\;BD+\\ 0.20\;CD+0.23\;A^{2}+0.68\;B^{2}-0.17\;C^{2}-0.26\;D^{2} \end{array}$$

(8) $$\begin{array}{c} DPPH=44.29+4.07\;A+2.34\;B+6.80\;C+2.81\;D-1.49\;AB-2.27\;AC-1.79\;AD-2.68\;BC+2.18\;BD-\\ 3.23\;CD+6.04\;A^{2}+5.78\;B^{2}+1.63\;C^{2}-1.40\;D^{2} \end{array}$$

(9) $$\begin{array}{c} FRAP=97.36+6.98\;A+8.22\;B+11.83\;C+5.92\;D+1.53\;AB-2.06\;AC+5.70\;AD-0.64\;BC-0.41\;BD+\\ 4.06\;CD+4.98\;A^{2}+11.71\;B^{2}+0.01\;C^{2}-6.88\;D^{2} \end{array}$$

Both the adequacy and goodness of fit of the generated regression models were then investigated through ANOVA and descriptive statistics (Table 4). The high F-values (13.91–181.14) and low *p*-values (<0.0001) obtained for the constructed models revealed that they were remarkably significant for all monitored responses. The insignificant lack of fit term (*p* > 0.05), observed for all models, confirmed the assumption of constant variances, which means that variance is a model-independent measure of pure error. At the same time, the high values obtained for the coefficients of determination (R^2^: 0.8070–0.9945) indicate a good agreement between the model and experimental results. Furthermore, the predicted R^2^ (R^2^_pred_) were in reasonable agreement with the adjusted R^2^ values (difference < 0.2), indicating a high degree of correlation between the experimental results and predicted values.

Based on the ANOVA results (Table 4), the significance of each independent variable in the responses was determined using the F-test and *p*-values. In general, as the absolute F-value increases and the *p*-value decreases, the corresponding factor becomes more notable, with variables having a *p*-value less than 0.05 (at 95% confidence level) considered significant for the response investigated. As observed, all responses (TPC, TFC, THA, TF, DPPH, and FRAP) were significantly influenced by the linear terms of the solvent-to-solid ratio (A), processing time (B), extraction temperature (C), and water content (D), except for TPC, which was not affected by the sonication time. All these factors exhibited a positive effect on the responses, except for the solvent-to-solid ratio, which demonstrated a negative effect on THA and TF. Among the interactive effects, AC and BC had significant effects on TPC; AB on TF; AB, AC, AD, BC, BD, and CD on DPPH; and AC, AD, and CD on the FRAP response, while TFC and THA were not influenced by partial cross coefficients. Regarding quadratic effects, the quadratic term of water content (D^2^) was statistically significant for all responses, except for THA. A^2^ and B^2^ demonstrated a significant impact on the TFC, TF, DPPH, and FRAP responses, while C^2^ only affected the DPPH variable.

#### 3.3.2. Response Surface Analysis

**Figure 4:**
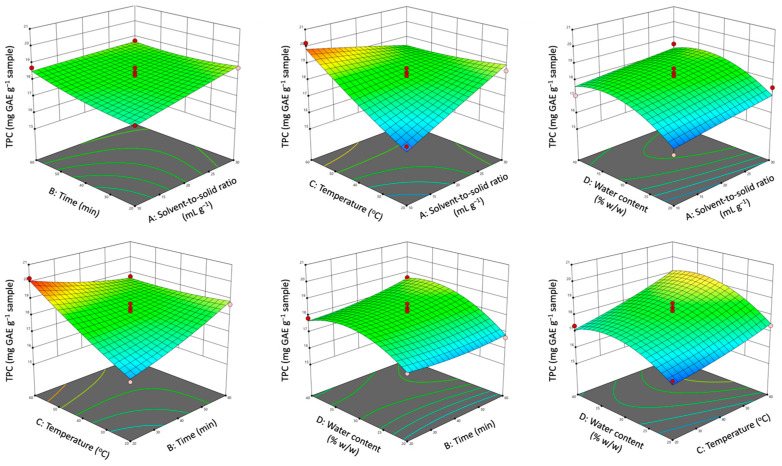
Response surface plots demonstrating the interactive effects of solvent-to-solid ratio, processing time, extraction temperature, and water content on TPC.

**Figure 5:**
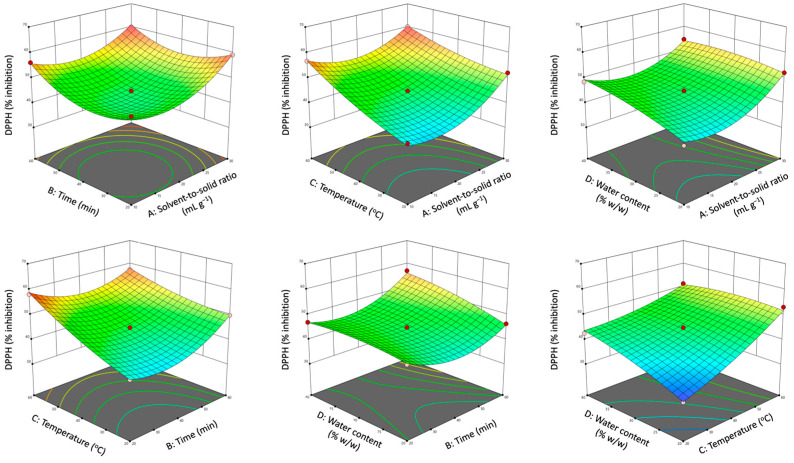
Response surface plots demonstrating the interactive effects of solvent-to-solid ratio, processing time, extraction temperature, and water content on DPPH.

Three-dimensional response surface plots were constructed based on the developed polynomial models to visually interpret the influence of independent variables and their interactions on the responses. Each graph illustrates the interactive effect of two independent variables while keeping the other two factors constant at their middle level. The effects of operational parameters on polyphenol yield and antioxidant activity in terms of TPC and DPPH are depicted in Figure 4 and Figure 5, while the remaining plots are presented in Figures S1–S4 (Supplementary Material).

As observed, the solvent-to-solid ratio had a positive effect on TPC, TFC, DPPH, and FRAP. Increased solvent-to-solid ratios resulted in increased concentration differences between the solid material and the solvent, improving the driving force of solute transfer from the plant cell to the extraction medium and, therefore, improving solute diffusivity [41]. However, an increase in solvent-to-solid ratios led to a decrease in THA and TF values. The reason for the maximum extraction yields of THA and TF at the lower limit of the solvent-to-solid ratio could be the stronger molecular interactions developed between the NaDES molecules and the phenolic components as a result of the increased contact surface area between the plant material and the solvent [31].

The recovery of TFC, THA and TF, and the antioxidant capacity of the extracts (DPPH and FRAP) were positively influenced by the extraction time. In particular, high values of responses occurred at the lower levels of processing time and even higher values at its upper limit. This is consistent with the literature which reports that the cavitation mechanism of UAE occurs in two main steps. In the first rapid step, referred to as the “washing step”, the dissolution of soluble bioactive components from the surface of the plant matrix into the extraction solvent takes place. The second step, which is the rate-determining step, involves the mass transfer of the soluble substances from the plant matrix to the solution phase by diffusion and osmotic processes [17]. As observed, the dissolution of the target components was achieved in the first 20 min, and then a slight decrease was observed for almost all monitored responses, probably due to the oxidation of the bioactive substances. A further increase in the extraction time resulted in an increase in the extraction yield and the antioxidant activity of the extracts. The increase in TPC, TFC, THA, TF, DPPH, and FRAP with the prolonging of extraction time is probably due to the rupture of the cell walls of the plant material due to the effect of acoustic cavitation, thus improving the release of intracellular components and the diffusivity of the target analytes [9]. It is worth mentioning here that although extraction time did not affect TPC values, its interaction with extraction temperature had a significant impact on the extractability of total polyphenols (TPC). In this case, increasing the temperature along with the extraction time may lead to thermal degradation of the sensitive bioactive substances, which is reflected by the low TPC yields obtained, indicating that extraction at elevated temperatures probably requires a shorter processing time. This was not the case for the remaining responses where increasing extraction time and temperature resulted in an improved extraction of antioxidants.

In general, an increase in temperature causes an increase in the kinetic energy of the solute particles and solvent molecules, thus increasing the diffusion rate of extraction [41]. In addition, increasing the kinetic energy of NaDES molecules results in a decrease in solvent viscosity, enhancing the interactions between the solid particles and the solvent components and improving the extraction efficiency of the process [42]. An increase in temperature also enhances the cavitation effect, facilitating the rupture of cell walls and the release of intracellular components during UAE [42]. As a result of all the aforementioned factors, increasing the extraction temperature had a positive effect on the extraction yield and antioxidant capacity.

Water content also had a significant effect on all responses. The addition of water to NaDES solutions can change both the polarity and viscosity of the solvent, leading to differences in extraction results [31]. In the present study, an increase in water content resulted in an initial rise in antioxidant extractability until a maximum yield was reached. After this maximum yield, however, the values of the responses gradually decreased with the addition of excess water, probably due to the weakening or breaking of the hydrogen bond network between the NaDES components [17].

#### 3.3.3. Multi-Response Optimization and Verification of the Model

The optimal extraction conditions were then determined using the desirability function method by maximizing polyphenols recovery (TPC, TFC, THA, and TF) and antioxidant capacity of the extracts (DPPH and FRAP). Based on the regression models, the optimal operational parameters for the UAE coupled with NaDES were as follows: a 10 mL g^−1^ solvent-to-solid ratio, a 60 min processing time, a 60 °C extraction temperature, and 33.8% (*w*/*w*) water content. These correspond to the maximum predicted values of 19.90 mg GAE g^−1^ sample for TPC, 7.49 mg CE g^−1^ sample for TFC, 4.54 mg CAE g^−1^ sample for THA, 9.49 mg QE g^−1^ sample for TF, 65.03% inhibition for DPPH, and 127.48 μmol TE g^−1^ sample for FRAP (desirability = 0.964). To verify the model’s validity, experiments were performed in triplicate under the determined optimal extraction conditions. The experimental values for TPC, TFC, THA, TF, DPPH, and FRAP were 19.68 ± 1.22 mg GAE g^−1^ sample, 7.40 ± 0.72 mg CE g^−1^ sample, 4.63 ± 0.20 mg CAE g^−1^ sample, 9.36 ± 0.56 mg QE g^−1^ sample, 65.80 ± 0.87% inhibition, and 126.62 ± 1.92 μmol TE g^−1^ sample, respectively. The good agreement between the experimentally obtained results and the theoretical values (percentage error < 5%, 1.09% for TPC, 1.42% for TFC, 1.83% for THA, 1.44% for TF, 1.19% for DPPH, and 0.68% for FRAP) confirms the good predictability and high accuracy of the developed models.

It is worth noting that the optimized extraction method yields significantly higher amounts of TPC than those reported in the literature. Singh et al. [43,44] reported TPC values in the range of 2.41–2.50 mg GAE g^−1^ sample for Colocasia leaf extracts obtained using a conventional organic solvent (aqueous methanol). Even lower TPC values (1.20 mg GAE g^−1^ sample) were reported by Lako et al. [45] during the phytochemical screening of Colocasia leaf extracts obtained using acetonitrile containing 4% (*v*/*v*) acetic acid as the extraction solvent. These differences highlight once again the higher efficiency of NaDESs compared with classical organic solvents in terms of polyphenol recovery. The lower values of total polyphenols (1.94–9.10 mg g^−1^) reported by Goncalves et al. and Ferreres et al. [1,2] for “giant white” and “red” taro varieties may be due to the higher selectivity of the analytical method used (HPLC-DAD), which is advantageous over spectrophotometric assays which are not selective and may often lead to an overestimation of TPC.

Regarding the flavonoid content of taro leaves, the proposed extraction method is also more effective in recovering flavonoids than previous non-optimized methods. The combined use of suitable solvent and ultrasound radiation results in an improved recovery of flavonoids; previous studies found significantly lower amounts of TFC (1.54 mg CE g^−1^ sample) [45]. Lako et al. [45] chromatographically quantified the flavonols present in Colocasia leaf extract and reported a value of 3 mg per 100 g for total flavonols, which is again much lower than the TF value determined for extracts obtained using UAE coupled with NaDES. In regard to antioxidant activity, a comparative study is considered extremely difficult as different concentrations of extracts are used to determine the DPPH inhibitory activity of taro leaves, while for the FRAP assay, there is only one study in the literature where the results are expressed using a different reference standard (1679.9 μΜ ferrous sulfate g^−1^) [19].

### 3.4. Protective Effects of Taro Extract against H_2_O_2_-Induced DNA Damage

Once the optimal extract was obtained (under the optimum UAE conditions: 10 mL g^−1^ solvent-to-solid ratio, 60 min processing time, 60 °C extraction temperature, and 33.8% (*w*/*w*) water content), it was further investigated whether it could protect cellular DNA in oxidative stress conditions. For that purpose, we used an in vitro cell culture system consisting of a human T-lymphoma cell line (Jurkat cells). The cultured cells were preincubated for 30 min with or without the indicated concentrations of the taro extract before being exposed to oxidative stress in the form of continuously generated H_2_O_2_. The formation of single-strand breaks in nuclear DNA was estimated by the single-cell gel electrophoresis methodology (comet assay) as described in Section 2.

**Figure 6:**
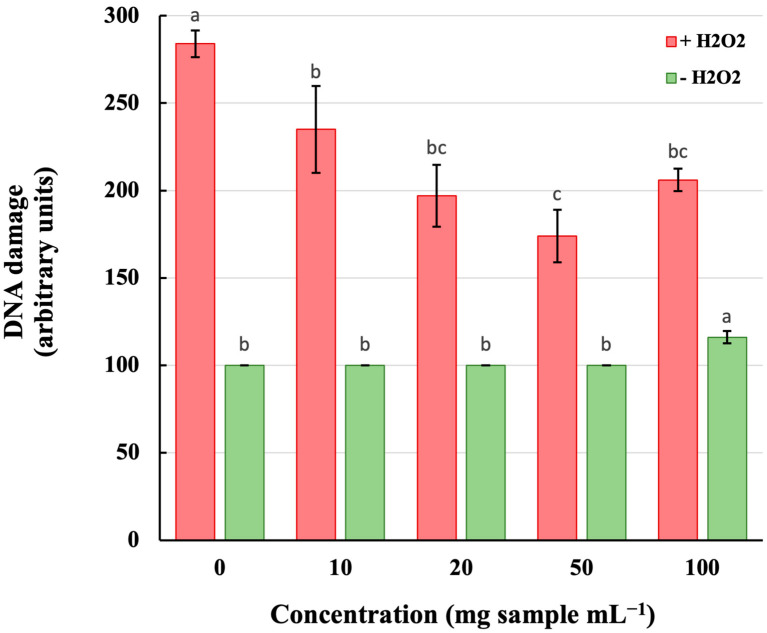
Protection offered by taro extracts against H_2_O_2_-induced DNA damage. Jurkat cells (150,000 cells per 100 μL) were preincubated for 30 min with the indicated concentrations of the taro extract (red bars) before being exposed for 15 min to continuously generated H_2_O_2_ (10 μM H_2_O_2_ per min) by the action of the glucose oxidase (G.O.) enzyme (green bars). DNA single-strand breaks were evaluated by the single-cell gel electrophoresis method (or comet assay) and expressed in arbitrary units as described in Materials and Methods. Each point represents the mean of three different experiments performed in duplicate. Different lowercase letters for each colored bar indicate significant differences (*p* < 0.05) according to Duncan’s multiple range test.

As shown in Figure 6, the taro extract was tested at concentrations ranging from 10 to 100 mg of sample per mL of cell culture. Preincubation of Jurkat cells with the taro extract before the addition of H_2_O_2_ diminished the formation of single-stranded DNA damage. Particularly, at the concentrations of 10, 20, and 50 mg/mL, the extract attenuated H_2_O_2_-induced DNA damage in a dose-dependent manner, reaching ~60% protection at the concentration of 50 mg/mL (red bars). Additionally, in these concentrations, the taro extract showed no cytotoxic effects (green bars). At the concentration of 100 mg/mL, however, the extract in the absence of H_2_O_2_ exerted DNA-damaging effects (green bars) and exhibited decreased protection against H_2_O_2_-induced DNA damage (red bars).

### 3.5. UPLC/ESI-Q-TOF MS Profiling of Phenolic Components of Taro Extract

The polyphenolic composition of the taro leaf extract, which was obtained using the optimal extraction parameters, was elucidated using UPLC/ESI-Q-TOF-MS. Table 5 summarizes the phenolic constituents of the taro leaf extract. The UPLC/ESI-Q-TOF-MS analysis allowed the tentative assignment of 12 phenolic compounds, classified into three main classes: flavones, flavonols, and caffeic acid derivatives. Flavones represent the main group of phenolic substances identified in the optimum extract of taro leaves, followed by flavonols and caffeic acid derivatives.

The chromatographic analysis revealed the presence of eight flavone derivatives in the taro leaf extract. Previous studies have also confirmed the presence of apigenin 6,8-di-C-hexoside, [1,2], isoorientin [1,5,20], luteolin 7-*O*-rutinoside [20], isovitexin [1,5], and chrysoeriol 7-*O*-hexoside [2] in leaf extracts obtained using conventional organic solvents. Among the flavonols, rutin was detected in a previous study, together with quercetin and kaempferol aglycones, demonstrating a similar polyphenolic pattern to that obtained in the present work [6]. Although caffeic acid, gallic acid, chlorogenic acid, coumaric acid, trans-cinnamic acid, and ellagic acid were detected in taro leaf extracts in previous studies [6,19], the optimal extract contains only dicaffeic acid. The phenolic profile of the taro leaf extract obtained using NaDES as an extraction medium was dominated by flavonoids and, in particular, flavones and flavonols, with a minor contribution of hydroxycinnamic acids. The NaDES components likely form stronger hydrogen bond networks with these substances, resulting in their higher extractability.

## 4. Conclusions

*Colocasia esculenta* L. leaves are considered a residue of taro cultivation and are discarded as waste to the environment, despite their valuable phytochemical composition. Their valorization to obtain value-added substances for medicinal, food, and cosmetic applications was the aim of this work. An eco-friendly and sustainable extraction procedure was developed for the effective recovery of taro leaf antioxidants using NaDESs coupled with UAE. Sixteen different NaDESs were prepared, and among them, the NaDES based on Bet and EtGl proved to be the most effective solvent. After selecting the most suitable solvent, the UAE operational parameters were optimized by the maximization of the phenolic content and antioxidant activity of the extracts using RSM. Multi-response optimization suggested a solvent-to-solid ratio of 10 mL g^−1^, a processing time of 60 min, an extraction temperature of 60 °C, and a water content of 33.8% (*w*/*w*) as optimal extraction parameters. Under these optimum conditions, a TPC of 19.68 ± 1.22 mg GAE g^−1^ sample, a TFC of 7.40 ± 0.72 mg CE g^−1^ sample, a THA of 4.63 ± 0.20 mg CAE g^−1^ sample, and a TF of 9.36 ± 0.56 mg QE g^−1^ sample were achieved. The leaf extract also demonstrated a strong radical scavenging activity against DPPH (65.80 ± 0.87%) and a high FRAP (126.62 ± 1.92 μmol TE g^−1^ sample). The UPLC/ESI-QTOF-MS analysis of the optimum extract revealed the predominance of flavones in the extract. Furthermore, the optimum extract protected human cultured cells against oxidative stress-induced DNA damage. The findings of the present work suggest that the residues of taro cultivation can potentially be an important and readily available source of polyphenolic antioxidants.

## Figures and Tables

**Table 1:** Viscosity and pH values of prepared natural deep eutectic solvents (NaDESs).

NaDES	Molar Ratio (HBA:HBD)	NaDES Category	Water Content			
			20% *w*/*w*		40% *w*/*w*	
			Viscosity (cP)	pH	Viscosity (cP)	pH
ChCl:EtGl	1:2	Polyol-based	35	7.80	24	6.76
ChCl:PrGl	1:2		51	7.80	30	6.84
ChCl:Gly	1:2		69	7.07	31	6.08
ChCl:LA	1:1	Acid-based	56	1.26	32	1.13
ChCl:CA	1:1		231	0.40	39	0.16
ChCl:Fru	1:1	Sugar-based	395	5.85	36	5.02
ChCl:Glc	1:1		520	6.21	38	5.57
ChCl:Suc	1:1		3100	6.25	56	5.23
Bet:EtGl	1:2	Polyol-based	56	8.54	29	7.46
Bet:PrGl	1:2		80	8.51	34	7.43
Bet:Gly	1:2		125	8.32	38	6.86
Bet:LA	1:1	Acid-based	122	4.36	36	3.53
Bet:CA	1:1		1383	2.19	44	2.24
Bet:Fru	1:1	Sugar-based	2517	7.45	52	5.88
Bet:Glc	1:1		4730	8.23	67	6.74
Bet:Suc	1:1		17,360	7.91	78	5.56

ChCl: choline chloride, Bet: betaine, EtGl: ethylene glycol, PrGl: propylene glycol, Gly: glycerol, LA: lactic acid, CA: citric acid, Fru: fructose, Glc: glucose, Suc: sucrose.

**Table 2:** Natural and coded levels of independent variables used in three-level, four-factor Box–Behnken design (BBD).

Independent Variable	Symbol	Factor Level		
		Low (−1)	Medium (0)	High (1)
Solvent-to-solid ratio (mL g^−1^)	A	10	20	30
Processing time (min)	B	20	40	60
Extraction temperature (°C)	C	20	40	60
Water content (%, *w*/*w*)	D	20	30	40

**Table 3:** BBD of the independent variables in their actual and coded values and experimentally obtained values of the investigated responses.

Run	Independent Variables				Responses					
	A Solvent-to-Solid Ratio (mL g^−1^)	B Processing Time (min)	C Extraction Temperature (°C)	D Water Content (%, *w*/*w*)	Y_1_ TPC (mg GAE g^−1^ Sample)	Y_2_ TFC (mg CE g^−1^ Sample)	Y_3_ THA (mg CAE g^−1^ Sample)	Y_4_ TF (mg QE g^−1^ Sample)	Y_5_ DPPH (% Inhibition)	Y_6_ FRAP (μmol TE g^−1^ Sample)
1	10 (−1)	40 (0)	40 (0)	40 (+1)	17.08	6.22	3.99	7.19	48.52	87.76
2	20 (0)	20 (−1)	40 (0)	20 (−1)	16.84	5.90	2.90	6.27	45.20	87.52
3	20 (0)	40 (0)	40 (0)	30 (0)	18.46	6.65	3.23	6.37	43.11	96.50
4	30 (+1)	40 (0)	40 (0)	40 (+1)	18.56	7.03	2.72	6.00	54.62	112.43
5	10 (−1)	40 (0)	20 (−1)	30 (0)	16.37	6.04	4.06	6.97	39.65	81.48
6	20 (0)	60 (+1)	20 (−1)	30 (0)	18.67	6.64	3.54	6.80	49.86	104.26
7	30 (+1)	40 (0)	40 (0)	20 (−1)	17.57	6.82	2.72	5.18	52.17	89.10
8	20 (0)	20 (−1)	20 (−1)	30 (0)	16.37	6.04	2.56	5.78	39.77	87.08
9	10 (−1)	60 (+1)	40 (0)	30 (0)	18.72	7.15	4.07	8.76	56.21	114.57
10	20 (0)	40 (0)	20 (−1)	40 (+1)	17.35	5.71	2.94	5.75	42.59	81.89
11	20 (0)	40 (0)	60 (+1)	40 (+1)	18.97	7.12	3.80	7.21	51.33	113.17
12	20 (0)	40 (0)	40 (0)	30 (0)	17.78	6.37	3.07	6.15	44.94	97.11
13	20 (0)	60 (+1)	40 (0)	40 (+1)	18.67	7.05	4.07	7.88	56.98	116.51
14	20 (0)	60 (+1)	60 (+1)	30 (0)	18.72	7.52	4.03	7.75	57.51	127.14
15	30 (+1)	40 (0)	20 (−1)	30 (0)	18.57	6.69	2.28	5.18	52.17	100.04
16	30 (+1)	20 (−1)	40 (0)	30 (0)	18.72	6.82	2.38	6.84	59.23	112.64
17	20 (0)	40 (0)	60 (+1)	20 (−1)	17.37	6.78	2.84	5.92	53.13	93.14
18	10 (−1)	40 (0)	60 (+1)	30 (0)	20.20	6.78	4.16	7.92	56.77	109.23
19	20 (0)	40 (0)	40 (0)	30 (0)	18.72	6.34	3.23	6.76	44.17	98.42
20	30 (+1)	60 (+1)	40 (0)	30 (0)	18.72	7.62	3.52	6.37	59.98	131.80
21	20 (0)	40 (0)	40 (0)	30 (0)	18.27	6.71	3.19	6.25	44.94	98.03
22	20 (0)	40 (0)	20 (−1)	20 (−1)	16.37	5.49	2.56	5.28	31.48	78.08
23	10 (−1)	40 (0)	40 (0)	20 (−1)	15.90	6.04	3.12	6.83	38.90	87.24
24	10 (−1)	20 (−1)	40 (0)	30 (0)	17.53	6.55	3.56	7.53	49.51	101.55
25	20 (0)	40 (0)	40 (0)	30 (0)	17.81	6.41	3.51	6.46	44.27	96.72
26	20 (0)	20 (−1)	60 (+1)	30 (0)	20.20	7.00	3.56	7.03	58.13	112.52
27	30 (+1)	40 (0)	60 (+1)	30 (0)	18.14	7.56	2.93	5.81	60.22	119.56
28	20 (0)	20 (−1)	40 (0)	40 (+1)	17.85	6.42	3.15	6.26	47.02	100.00
29	20 (0)	60 (+1)	40 (0)	20 (−1)	16.67	6.92	3.12	6.92	46.43	105.66

TPC: total phenolic content, TFC: total flavonoid content, THA: total hydroxycinnamic acids, TF: total flavonols, DPPH: 2,2-diphenyl-1-picrylhydrazyl radical scavenging activity, FRAP: ferric reducing antioxidant power.

**Table 4:** Analysis of variance (ANOVA) and descriptive statistics of the fitted models.

Term	TPC		TFC		THA		TF		DPPH		FRAP	
	F-Value	*p*-Value	F-Value	*p*-Value	F-Value	*p*-Value	F-Value	*p*-Value	F-Value	*p*-Value	F-Value	*p*-Value
Model	13.91	<0.0001	17.64	<0.0001	25.09	<0.0001	22.12	<0.0001	97.36	<0.0001	181.14	<0.0001
A	10.58	0.0058	38.81	<0.0001	50.23	<0.0001	124.68	<0.0001	180.40	<0.0001	279.61	<0.0001
B	3.77	0.0727	47.33	<0.0001	22.04	<0.0001	29.49	<0.0001	59.74	<0.0001	387.89	<0.0001
C	52.31	<0.0001	103.32	<0.0001	13.91	0.0010	44.50	<0.0001	503.21	<0.0001	803.09	<0.0001
D	31.96	<0.0001	6.82	0.0205	14.18	0.0009	19.53	0.0006	86.12	<0.0001	201.16	<0.0001
AB	2.27	0.1537	0.3065	0.5886	-	-	11.31	0.0046	8.03	0.0133	4.50	0.0522
AC	28.92	<0.0001	0.1120	0.7428	-	-	0.4122	0.5312	18.72	0.0007	8.10	0.0129
AD	0.0565	0.8156	0.0073	0.9331	-	-	0.8165	0.3815	11.63	0.0042	62.21	<0.0001
BC	22.73	0.0003	0.0530	0.8212	-	-	0.3600	0.5581	26.03	0.0002	0.7839	0.3909
BD	1.58	0.2300	1.28	0.2775	-	-	3.69	0.0754	17.33	0.0010	0.3176	0.5819
CD	0.6191	0.4445	0.1029	0.7531	-	-	2.57	0.1313	37.81	<0.0001	31.47	<0.0001
A^2^	0.0104	0.9201	13.76	0.0023	-	-	5.40	0.0356	214.95	<0.0001	77.01	<0.0001
B^2^	1.38	0.2589	18.38	0.0008	-	-	46.75	<0.0001	196.62	<0.0001	425.75	<0.0001
C^2^	0.7590	0.3983	0.0354	0.8534	-	-	2.79	0.1169	15.57	0.0015	0.0001	0.9922
D^2^	31.87	<0.0001	10.41	0.0061	-	-	6.60	0.0223	11.59	0.0043	146.96	<0.0001
Lack of fit	0.8997	0.5964	1.08	0.5153	2.99	0.1485	1.24	0.4533	2.35	0.2125	3.79	0.1053
R^2^	0.9329		0.9464		0.8070		0.9567		0.9898		0.9945	
R^2^_adj_	0.8658		0.8927		0.7749		0.9135		0.9797		0.9890	
R^2^_pred_	0.7003		0.7521		0.7038		0.7953		0.9476		0.9706	

**Table 5:** Qualitative data from the UPLC/ESI-Q-TOF-MS analysis of the optimum taro leaf extract.

Compound	Molecular Formula	Neutral Mass (Da)	Observed *m*/*z* for [M-H]^−^	Mass Error (ppm)	Identified Polyphenol	Group
1	C_27_H_30_O_15_	594.158	593.151	−0.6	Vicenin-2(Apigenin 6,8-di-C-glucoside)	Flavone
2	C_26_H_28_O_15_	580.143	579.135	−0.8	Luteolin 7-*O*-(2-apiosyl-glucoside)	Flavone
3	C_26_H_28_O_14_	564.148	563.14	−0.3	Apioside (Apigenin 7-*O*-apiosyl-glucoside)	Flavone
4	C_27_H_30_O_16_	610.153	609.146	−0.4	Antoside	Flavonol
5	C_21_H_20_O_11_	448.101	447.093	0	Isoorientin (Luteolin 6-C-glucoside)	Flavone
6	C_18_H_14_O_8_	358.069	357.061	−0.3	5,5′-Dicaffeic acid	Hydroxycinnamic acid
7	C_27_H_30_O_16_	610.153	609.146	−0.7	Rutin (quercetin 3-rutinoside)	Flavonol
8	C_27_H_30_O_15_	594.158	593.151	−0.7	Luteolin 7-*O*-rutinoside	Flavone
9	C_21_H_20_O_10_	432.106	431.098	−0.1	Isovitexin (Apigenin 6-C-glucoside)	Flavone
10	C_27_H_30_O_15_	594.158	593.151	−0.1	Kaempferol 3-*O*-galactoside 7-*O*-rhamnoside	Flavonol
11	C_22_H_22_O_11_	462.116	461.109	−0.4	Chrysoeriol 7-*O*-glucoside	Flavone
12	C_16_H_12_O_6_	300.063	299.056	0.3	Chrysoeriol	Flavone

## Data Availability

All of the data is contained within the article and the supplementary materials.

## Supplementary Materials

The following supporting information can be downloaded at https://www.mdpi.com/article/10.3390/antiox12101801/s1: Figures S1–S4, Response surface plots demonstrating the interactive effects of solvent-to-solid ratio, processing time, extraction temperature, and water content in NaDESs on TFC, THA, TF, and FRAP.
