# Supplementary material for: Ultrasound-Assisted Extraction of Taro Leaf Antioxidants Using Natural Deep Eutectic Solvents: An Eco-Friendly Strategy for the Valorization of Crop Residues
Source: Antioxidants (Basel). 2023 Sep 26;12(10):1801. doi: 10.3390/antiox12101801 (PMC10604219; doi:10.3390/antiox12101801)
Supplement: Supplementary file 1 [file antioxidants-12-01801-s001.zip › antioxidants-2625223-supplementary.pdf]

Supplementary Material

Ultrasound-assisted extraction of taro leaf antioxidants using natural deep eutectic solvents: An eco-friendly strategy for the valorization of crop residues

Atalanti Christou*[1], Nikolaos A. Parisis [2], Themistoklis Venianakis [2], Alexandra Barbouti [3], Andreas G. Tzakos [2], Ioannis P. Gerothanassis [2], and Vlasios Goulas [1,*]

[1] Cyprus University of Technology, Department of Agricultural Sciences, Biotechnology and Food Science, Lemesos, 3603; atalanti.christou@cut.ac.cy (AC); vlasios.goulas@cut.ac.cy (VG)
[2] Department of Chemistry, Section of Organic Chemistry and Biochemistry, University of Ioannina, 45110 Ioannina, Greece, nikparis@gmail.com (NP); vethemis@gmail.com (TH); atzakos@uoi.gr (AT); igeoth@uoi.gr (IG)
[3] Department of Anatomy-Histology-Embryology, Faculty of Medicine, School of Health Sciences, University of Ioannina, 45110 Ioannina, Greece; abarbout@uoi.gr
* Correspondence: vlasios.goulas@cut.ac.cy; Tel.: +357-2500-2141 (V.G); atalanti.christou@cut.ac.cy (AC)

Table of figures:

Figure S1. Response surface plots demonstrating the interactive effects of solvent-to-solid ratio, processing time, extraction temperature, and water content on TFC. 3
Figure S2. Response surface plots demonstrating the interactive effects of solvent-to-solid ratio, processing time, extraction temperature, and water content on THA. 3
Figure S3. Response surface plots demonstrating the interactive effects of solvent-to-solid ratio, processing time, extraction temperature, and water content on TF. 4
Figure S4. Response surface plots demonstrating the interactive effects of solvent-to-solid ratio, processing time, extraction temperature, and water content on FRAP. 4

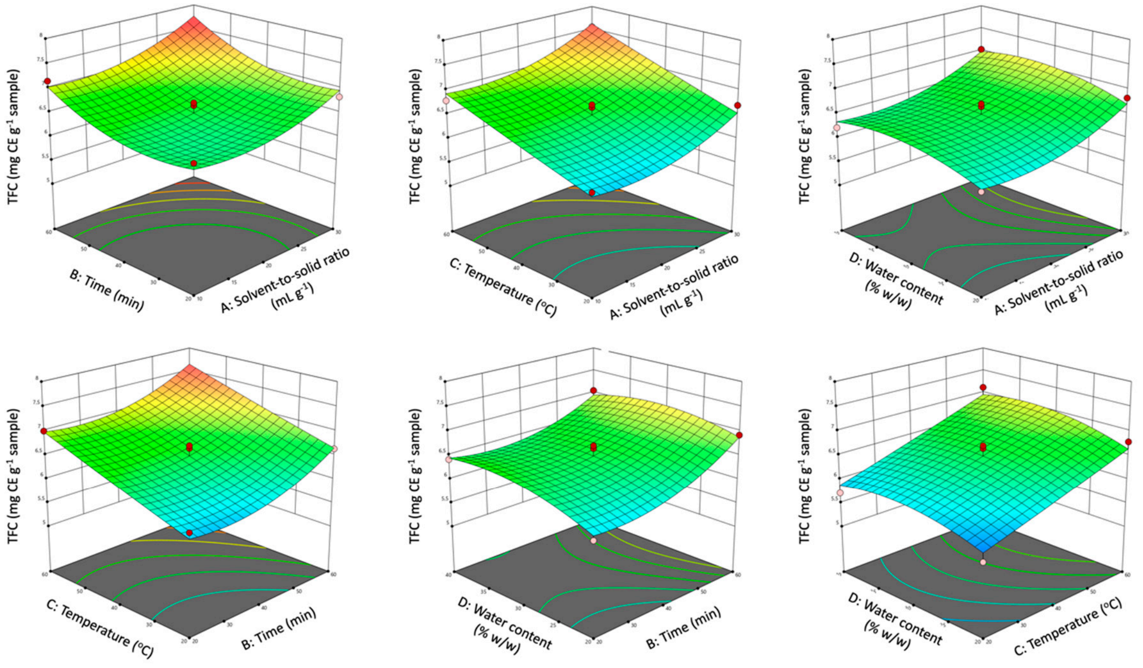

Figure S1. Response surface plots demonstrating the interactive effects of solvent-to-solid ratio, processing time, extraction temperature, and water content on TFC.

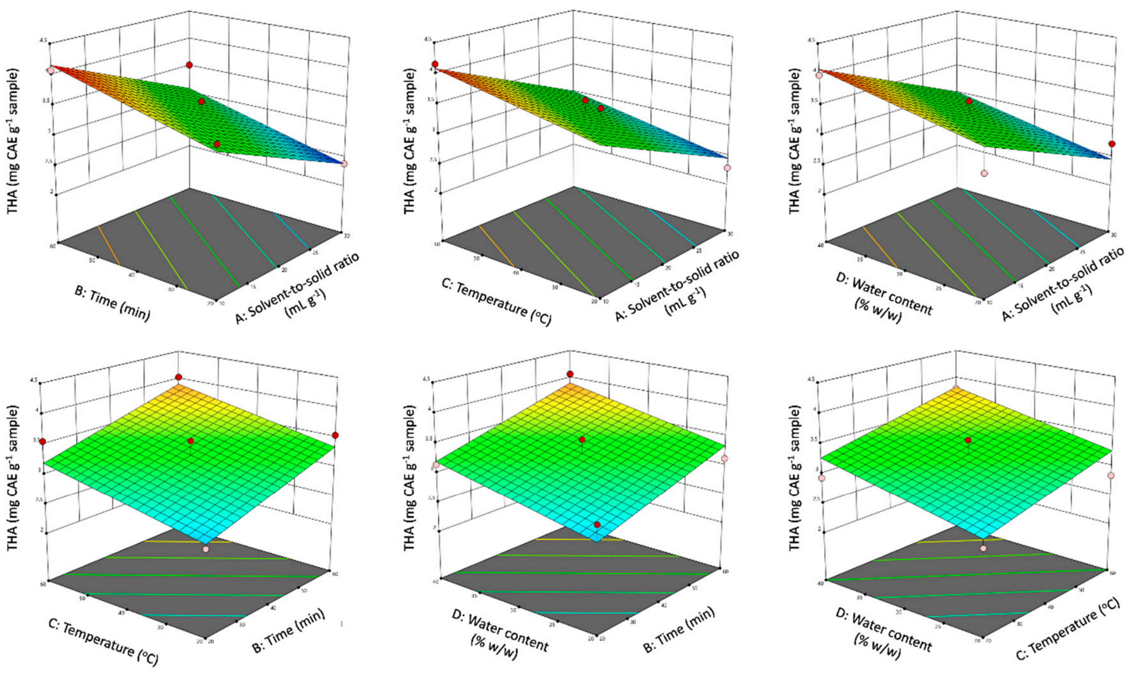

Figure S2. Response surface plots demonstrating the interactive effects of solvent-to-solid ratio, processing time, extraction temperature, and water content on THA.

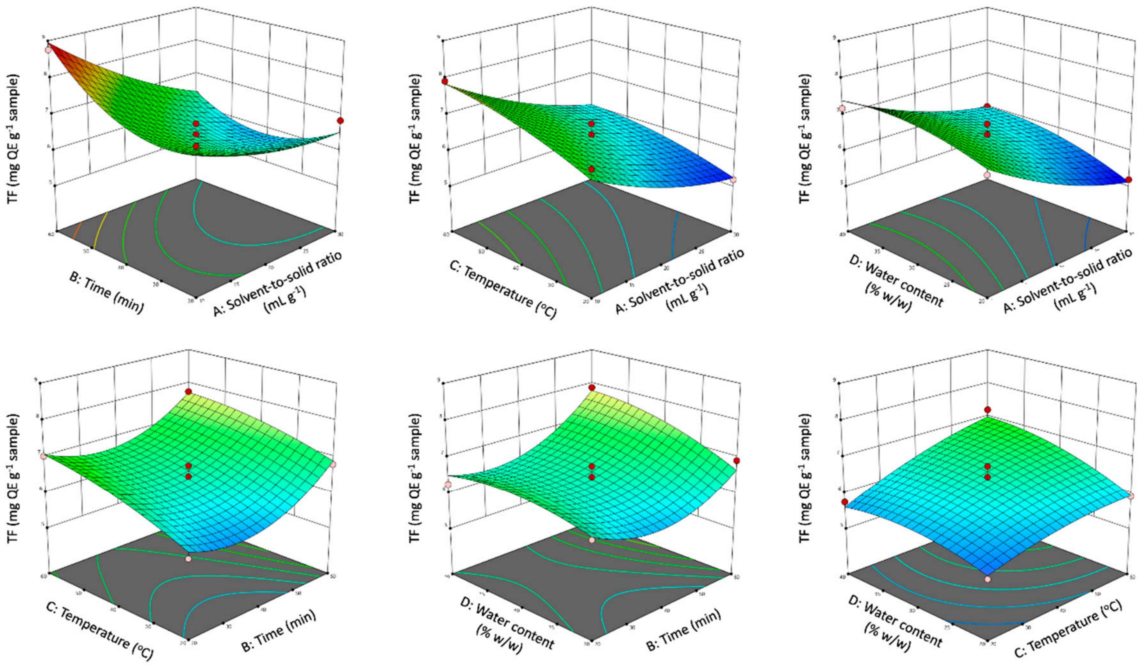

Figure S3. Response surface plots demonstrating the interactive effects of solvent-to-solid ratio, processing time, extraction temperature, and water content on TF.

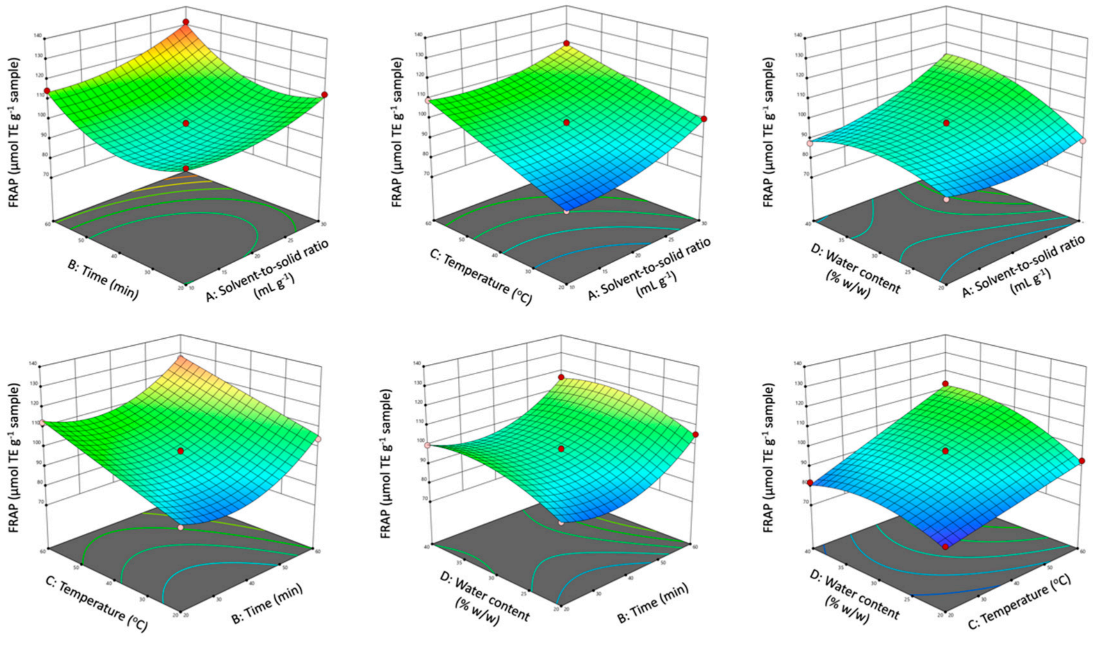

Figure S4. Response surface plots demonstrating the interactive effects of solvent-to-solid ratio, processing time, extraction temperature, and water content on FRAP.
